# Parameter adaptations during phenotype transitions in progressive diseases

**DOI:** 10.1186/1752-0509-5-174

**Published:** 2011-10-26

**Authors:** Christian A Tiemann, Joep Vanlier, Peter AJ Hilbers, Natal AW van Riel

**Affiliations:** 1 Department of BioMedical Engineering, Eindhoven University of Technology, Den Dolech 2, Eindhoven, 5612 AZ, The Netherlands; 2 Netherlands Consortium for Systems Biology, University of Amsterdam, Science Park 904, Amsterdam, 1098 XH, The Netherlands

## Abstract

**Background:**

The study of phenotype transitions is important to understand progressive diseases, e.g., diabetes mellitus, metabolic syndrome, and cardiovascular diseases. A challenge remains to explain phenotype transitions in terms of adaptations in molecular components and interactions in underlying biological systems.

**Results:**

Here, mathematical modeling is used to describe the different phenotypes by integrating experimental data on metabolic pools and fluxes. Subsequently, trajectories of parameter adaptations are identified that are essential for the phenotypical changes. These changes in parameters reflect progressive adaptations at the transcriptome and proteome level, which occur at larger timescales. The approach was employed to study the metabolic processes underlying liver X receptor induced hepatic steatosis. Model analysis predicts which molecular processes adapt in time after pharmacological activation of the liver X receptor. Our results show that hepatic triglyceride fluxes are increased and triglycerides are especially stored in cytosolic fractions, rather than in endoplasmic reticulum fractions. Furthermore, the model reveals several possible scenarios for adaptations in cholesterol metabolism. According to the analysis, the additional quantification of one cholesterol flux is sufficient to exclude many of these hypotheses.

**Conclusions:**

We propose a generic computational approach to analyze biological systems evolving through various phenotypes and to predict which molecular processes are responsible for the transition. For the case of liver X receptor induced hepatic steatosis the novel approach yields information about the redistribution of fluxes and pools of triglycerides and cholesterols that was not directly apparent from the experimental data. Model analysis provides guidance which specific molecular processes to study in more detail to obtain further understanding of the underlying biological system.

## Background

Cardiovascular and metabolic diseases such as diabetes mellitus and metabolic syndrome are progressive in time [1-5]. Progressive diseases are often being studied by experimentally comparing different states: a control state representing a healthy phenotype, and one or more adapted states representing phenotypes of certain stages of the disease. Experimentally observed differences between phenotypes provide information about biological processes that are involved in the pathogenesis. Most research is carried out using mouse models, having many practical advantages such as short generation times, reduced genetic variation, and the possibility to apply gene manipulation technology [6-8]. For instance, the genetic leptin-deficient (ob/ob) or leptin-resistant (db/db) mouse are frequently used to study metabolic pathologies, e.g., obesity, insulin resistance, and diabetes [9-12]. A challenging task is to explain phenotypical characteristics and the progression of phenotype transitions in terms of adaptations in molecular components and interactions in underlying biological systems. This is especially the case for the study of progressive diseases in which multiple processes, operating on various length and timescales, are altered.

In systems biology mathematical modeling is applied to integrate different sources of experimental data of a phenotype and to investigate the complex interactions of underlying biological systems [13-19]. However, several issues complicate the simulation and prediction of molecular adaptations associated with progressive diseases. One problem is to cover large differences in timescales. Computational models in molecular systems biology are typically constructed to simulate processes on a single timescale. These range from seconds in signal transduction and metabolic network models to hours for genetic networks [20-24]. On the other hand, progressive diseases often comprise of a combination of these processes and typically develop over a time span of years in humans. Another issue is that mathematically describing progressive adaptations could become unfeasible when sufficient information of the underlying biological system, such as network structure, molecular concentrations and fluxes, as well as their interaction mechanisms, is lacking.

In the present work, we propose a novel computational approach to analyze molecular adaptations in a biological system to overcome these problems. We use mathematical modeling to quantitatively integrate metabolic data of different phenotypes and subsequently exploit this mathematical framework to analyze which molecular processes have changed and are collectively responsible for the shift between phenotypes. This information is obtained by identifying the progression of necessary parameter changes required for the model to be consistent with the experimental data of these phenotypes. These changes in parameters reflect progressive adaptations at the transcriptome and proteome level, which occur at larger timescales than the metabolic processes. The approach involves consecutive steps of data gathering, model development, and parameter estimation, which will be discussed in detail. An advantage of our approach is that mathematical models containing processes at any timescale of interest can be used, while their long-term adaptations are captured by identifying necessary parameter changes. This enables us to study long-term aspects of short-term processes. Furthermore, in cases when the amount of information of the underlying biological system is limited, our approach could provide a means to describe adaptations in molecular processes without the necessity to develop detailed kinetic models of the modulating mechanisms. For instance, if one is interested in studying a metabolic pathway which is adapting due to activation of a signal transduction pathway, the modulating effects can be captured by identifying necessary changes in the metabolic pathway parameters rather than developing a mathematical model that includes an explicitly modeled signal transduction pathway. The approach, which is applicable to a multitude of biological systems, is demonstrated on the basis of a case involving the activation of the liver X receptor (LXR), a promising drug target for atherosclerotic therapies [25,26].

The family of liver X receptors (LXR*α *and LXR*β*) is involved in the control of cellular lipid metabolism. LXRs, when ligand-activated by oxysterols, heterodimerize with the retinoid X receptor (RXR) and bind to LXR responsive elements on the DNA [27], where they induce the transcription of lipogenic genes such as SREBP-1C, FAS, ABCA1, and ACC1. Hereby they modulate the control of cholesterol, fatty acid, triglyceride, and lipoprotein metabolism. As a consequence, LXRs have emerged as promising drug targets for pharmacological LXR agonists to treat metabolic diseases like atherosclerosis and type 2 diabetes [28]. In rodents it has been shown that synthetic LXR agonists (T0901317, GW3965, and WAY252623) promote cellular cholesterol efflux, transport, and excretion, herewith halting the progression of atherosclerosis. However, pharmacological LXR activation also induces hepatic steatosis and promotes the secretion of enlarged atherogenic very-low-density-lipoprotein (VLDL) particles by the liver, complicating the clinical application of LXR agonists [29,30]. In the present study, we applied our computational approach to determine which metabolic processes change upon LXR activation, and identify the progression of molecular adaptations that collectively result in a shift of phenotype (wild-type versus LXR activated state). Parameters that are critical to the phenotype transition are considered candidates as biomarkers for disease diagnosis, treatment, or even prevention.

## Methods

Several theoretical sections are presented describing the methodology of the computational approach, which involves consecutive steps of data gathering, model development, and various parameter estimation steps.

### Model development

The computational approach is developed to analyze progressively adapting biological systems that are modeled using a system of (non)linear ordinary differential equations (ODEs):

(1) $$ẋ\left(t,\theta \right)=f\left(x\left(t,\theta \right),\theta ,u\left(t\right)\right)\;\text{with}\;x\left(t_{0},\theta \right)=x_{0}$$

where  ẋ is a vector of flux descriptions of molecular species *x*, which are given by a set of functions *f*, that in turn depend on kinetic parameters *θ *and model inputs *u*. The initial concentrations of molecular species *x *are given by *x*_0_. The vector of model outputs *y *is given by:

(2) y(t,θ)=g(x(t,θ),θ,u(t))

which is described by a set of functions *g *depending on molecular species *x*, kinetic parameters *θ*, and model inputs *u*.

### Simulating the biological system of phenotype A

Once a network topology of molecular species and corresponding flux descriptions are defined, values for the kinetic parameters *θ *have to be specified in order to perform simulations and make predictions. One way of determining parameter values is to directly measure them. However, this could become impractical when it is not possible to perform the necessary experiments, or model parameters do not have a well-defined physiological meaning, e.g., when multiple processes are lumped into a single model parameter. Another way to obtain parameter values, which was employed here, is to estimate them by minimizing the difference between experimental data and corresponding model simulations [31]. The amount of experimental data is usually limited compared to the number of parameters, resulting in non-unique solutions for the model parameters. Hence, multiple parameter sets exist that adequately describe the experimental data. Conversely, model predictions of unmeasured molecular species might potentially vary greatly depending on the chosen parameter set. To assess the uncertainty associated with model predictions, differences between feasible parameter sets must be examined [32-34]. A large-scale parameter estimation protocol was employed to capture multiple parameter sets describing the biological system of phenotype A. First, parameter regions were identified that are most likely to describe the experimental data. To this end, a collection of one hundred million parameter sets was sampled from a log-uniform distribution, capturing a parameter range of twelve orders of magnitude (10^-6 ^to 10^6^). For each parameter set a simulation to steady-state was carried out. Subsequently, the weighted sum of squared errors *X_d _*(*θ*) between the experimental data of phenotype A and corresponding steady-state model outputs were determined:

(3) $$X_{d}\left(\theta \right)=\overset{N}{\underset{i=1}{\sum }}{\left(\frac{y_{i}\left(\theta \right)-d_{i}^{A}}{\sigma_{i}^{A}}\right)}^{2}$$

where *N *is the number of measurements, *d^A ^*and *σ^A ^*respectively the means and standard deviations of the experimental data of phenotype A, and *y *the corresponding model outputs. Furthermore, a Monte Carlo approach was employed to account for experimental uncertainties. Each simulation a different realization for *d^A ^*was used. It was assumed that the experimental data is Gaussian distributed with means *μ^A ^*and standard deviations $\sigma^{A}\left(d_{i}^{A}=N\left(\mu_{i}^{A},\sigma_{i}^{A}\right)\right)$. Subsequently, the ten thousand best parameter sets (lowest *X_d _*values) were selected and optimized to describe the experimental data, by applying a weighted non-linear least squares algorithm that minimizes *X_d _*(*θ*):

(4) $$\hat{\theta }=\arg\;\underset{\theta }{\min}X_{d}\left(\theta \right)$$

where $\hat{\theta }$ represents the optimized parameter set. An optimized parameter set was acceptable if corresponding model outputs were in the confidence intervals of the experimental data. A significance level of 0.05, adjusted by Bonferroni correction to account for the number of comparisons being performed (number of model outputs), was used [35].

### Identification of molecular adaptations from phenotype A to phenotype B

#### Parameter estimation to describe phenotype B

The mathematical model together with the collection of acceptable parameter sets, represents the biological system of phenotype A. Molecular processes that are responsible for the transition of the biological system from phenotype A to phenotype B, are determined by identifying kinetic parameters that necessarily have to change in order to describe the biological system of phenotype B. A first approach could be to repeat the large-scale parameter estimation protocol, employed on phenotype A, for phenotype B. However, apart from being computationally expensive, comparing parameter sets from different phenotypes with each other is problematic, as they are obtained independently from each other. For instance, in the case when multiple separate minima exist, it would not be possible to know which realization of phenotype A is the reference for a specific realization of phenotype B. However, the fact that phenotype B originates from phenotype A could be used to address latter problem. The acceptable parameter sets from phenotype A could be used as initial values and reoptimized by once more applying a weighted non-linear least squares algorithm, minimizing *X_d_*(*θ*) with respect to the experimental data of phenotype B. Subsequently, necessary parameter adaptations can be identified which are responsible for the change of phenotype.

#### Iterative data integration and parameter estimation

Parameter adaptations describing a phenotype transition are often not unique. For instance, in order to increase a specific molecular concentration, corresponding production and degradation parameters can be changed in infinitely many different ways to accomplish this. Here, we assume that adaptations are minimal and proceed progressively in time. Therefore, the concept described in the previous section was extended to study progressively adapting biological systems, by defining artificial intermediate phenotypes. Hereto, the experimental data is interpolated from phenotype A to phenotype B in a number of steps. For instance, for a linear interpolation scheme this would imply *d^q ^*= (1 - *q*)*d^A ^*+ *qd^B^*, where *d^A ^*and *d^B ^*respectively represent the experimental data of phenotype A and B, and *q *a coordinate ranging from zero (completely phenotype A) to one (completely phenotype B). At each interpolation step the parameters are reoptimized in order to describe the newly interpolated data. The final values of the model states and parameters of the current optimization step are used as initial values for the next optimization step. This procedure is repeated until the final state representing phenotype B is reached and a parameter adaptation trajectory is obtained. The new objective function becomes as follows:

(5) $$X_{d}\left(\theta^{q}\right)=\overset{N}{\underset{i=1}{\sum }}{\left(\frac{y_{i}\left(\theta^{q}\right)-d_{i}^{q}}{\sigma_{i}^{q}}\right)}^{2}$$

Similar as in equation (3), for each parameter trajectory different realizations for *d^A ^*and *d^B ^*were used to account for experimental uncertainties.

#### Regularization of parameter adaptation trajectories

It is assumed that adaptations are minimal and proceed progressively in time. Therefore, the parameter estimation protocol was extended to avoid needless change of parameters, hereby identifying minimal parameter adaptations that are necessary to describe a phenotype transition. To this end, *X_d _*could be combined with a regularization term *X_r _*given by the sum of squared parameter changes. When changing a parameter is costly, it will be avoided if not necessary. The new objective function is given by:

(6) $$\begin{array}{llll} X\left(\theta^{q}\right) & =X_{d}\left(\theta^{q}\right)+\lambda X_{r}\left(\theta^{q}\right)\; & & \;\\ & =\overset{N}{\underset{i=1}{\sum }}{\left(\frac{y_{i}\left(\theta^{q}\right)-d_{i}^{q}}{\sigma_{i}^{q}}\right)}^{2}+\lambda \overset{M}{\underset{j=1}{\sum }}{\left(\frac{\theta_{j}^{q}-\theta_{j}^{0}}{\theta_{j}^{0}}\right)}^{2}\; & & \;\\ & \; & \end{array}$$

where *M *is the number of parameters, $\theta_{j}^{0}$ the initial parameter set representing phenotype A, and *λ *a constant determining the strength of the regularization term.

#### Consistency of parameter adaptation trajectories

The identification of parameter adaptation trajectories was performed for each acceptable parameter set, which gives information about the possible dispersion of parameter trajectories due to kinetic variations between the different acceptable parameter sets. However, given the uncertainties arising from experimental data and parameter estimates, the reliability of individual parameter trajectories is also a relevant topic to explore; is an observed trajectory consistent or is its path just a coincidental result? Given a certain parameter trajectory, it is important to analyze how reliable and consistent its path is to eventually draw conclusions about potential molecular adaptations that could have taken place. Therefore, the protocol described above was extended by not only determining parameter trajectories from phenotype A to phenotype B, but also backwards from phenotype B to phenotype A. A backward trajectory is obtained by interpolating the data from phenotype B to phenotype A, whilst reoptimizing the parameters. The final values of the model states and parameters obtained from the forward trajectory are used as initial values to calculate the backward trajectory. Furthermore, the reference parameter set $\theta_{j}^{0}$ (equation 6) is exchanged in this case by $\theta_{j}^{1}$ (the initial parameter set representing phenotype B) in order to regularize the backward trajectory. This process can be repeated an arbitrary number of steps, each time using the newly obtained values for the model states and parameters as initial values. The obtained parameter trajectories have been analyzed for consistency, which gives information regarding how well these adaptations are constrained by the data and can be predicted by the model. It must be noted that the calculation of backward trajectories is mainly a mathematical technique to assess the robustness of a specific solution. Hence, these trajectories do not necessarily have to exist physiologically.

## Results

We presented a computational approach to analyze molecular adaptations in a biological system evolving through various phenotypes, which is generically applicable to different biological systems. In this section, the computational approach is demonstrated by applying it to a case of liver X receptor induced hepatic steatosis.

### Experimental data

The acquisition of quantitative experimental data of different phenotypes is essential to gain insight in the progression of molecular adaptations in underlying biological systems. The available experimental data determines to a large extend the development of a mathematical model. The level of detail and precision at which certain biological processes can be integrated in a mathematical model, is determined by the selection of molecular species, as well as the type and quality of the measurements. With respect to the LXR case, several datasets of wild-type and T0901317 LXR activated C57BL/6J mice were obtained. Data was included containing measurements of hepatic triglyceride, free cholesterol, and cholesterylester levels, as well as plasma triglyceride, high-density-lipoprotein (HDL) cholesterol, total cholesterol, and free fatty acid levels in overnight-fasted mice [29]. Furthermore, data of nascent produced VLDL particles such as diameter, triglyceride/cholesterol composition ratio, and VLDL triglyceride production rate was used [29]. Data was included containing rate measurements of hepatic cholesterol production, hepatic cholesterol uptake via HDL, and cholesterol uptake by peripheral tissues [36]. Information about the deposition and production of hepatic triglycerides in cytoplasmic and microsomal fractions was included [37]. An overview of the obtained experimental data is included in Additional file 1.

### Computational model of hepatic lipid and plasma lipoprotein metabolism

**Figure 1:**
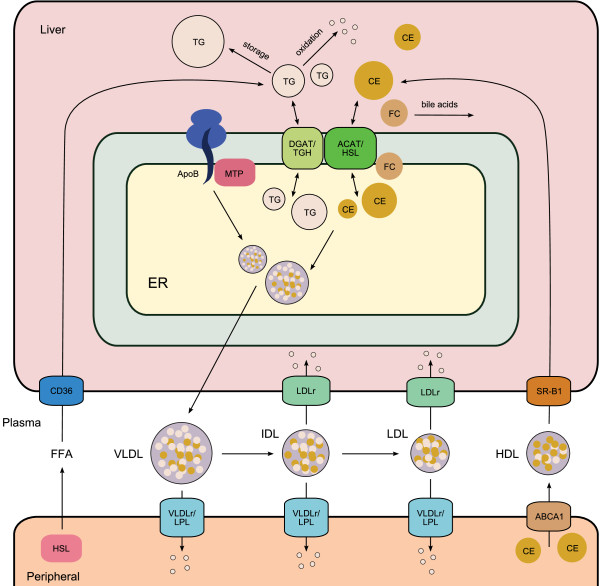
**Hepatic lipid and plasma lipoprotein metabolism**. The mathematical model has three compartments representing the liver, blood plasma, and peripheral tissues. The liver compartment includes reactions representing the production, utilization and storage of triglycerides and cholesterols, and the mobilization of these metabolites to the endoplasmic reticulum, where they are incorporated into nascent produced VLDL particles. The VLDL particles are secreted in the plasma compartment where they serve as nutrients for peripheral tissues. Remnant particles are taken up and cleared by the liver. The model furthermore includes the hepatic uptake of free fatty acids and reverse cholesterol transport via HDL. ABCA1, ATP-binding cassette transporter 1; ACAT, acyl-CoA: cholesterol acyltransferase; ApoB, apolipoprotein B; CE, cholesterylester; DGAT, diglyceride acyltransferase; ER, endoplasmic reticulum; FFA, free fatty acid; FC, free cholesterol; HDL, high-density-lipoprotein; HSL, hormone-sensitive lipase; IDL, intermediate density lipoprotein; LDL, low density lipoprotein; LDLr, low density lipoprotein receptor; LPL, lipoprotein lipase; MTP, microsomal triglyceride transfer protein; SR-B1, scavenger receptor class B1; TG, triglyceride; TGH, triglyceride hydrolase; VLDL, very low density lipoprotein; VLDLr, very low density lipoprotein receptor.

A mathematical multi-compartment model was constructed, based on the available experimental data, which integrates metabolic processes involved in hepatic lipid metabolism, as well as plasma lipoprotein metabolism (Figure 1). The mathematical model contains three compartments representing the liver, blood plasma, and periphery. The liver compartment includes reactions representing the production, utilization and storage of triglycerides and cholesterols. Furthermore, the model includes the mobilization of these metabolites to the endoplasmic reticulum, where they are incorporated into nascent produced VLDL particles. These VLDL particles are subsequently secreted in the plasma compartment where they serve as nutrients for peripheral tissues, e.g., muscle, heart, and adipose tissue. Remnant particles are taken up and cleared by the liver. The model furthermore includes the hepatic uptake of free fatty acids and the reverse transport of cholesterol via HDL. The model size and complexity of the reaction equations was kept to a minimum to preserve feasibility of model analyses and parameter estimation. The model developed contains eight molecular species *x *and twenty-two kinetic parameters *θ*. The flux descriptions *f *are all based on mass action kinetics. A description of the mathematical model, including equations, is presented in Additional file 1. Furthermore, an implementation of the model is available in SBML format (Additional file 2).

### Simulating the wild-type mouse

**Figure 2:**
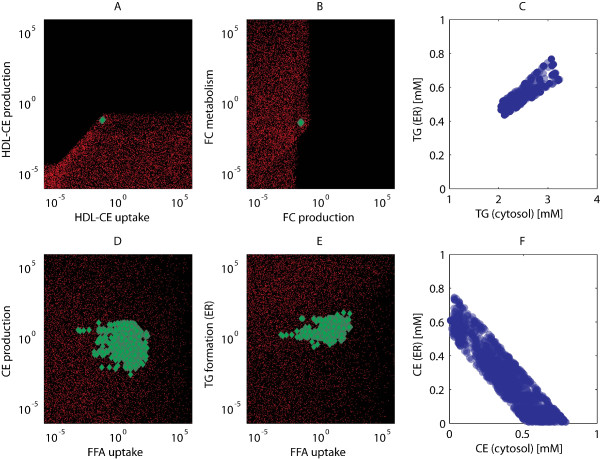
**Parameter scatter plots and predictions**. Several parameters are very constrained by the data and have a well defined value (A and B), whereas others show a larger spread of possible values (D and E). The black dots represent the 10^8 ^initial sampled parameter values (individual dots not visible), whereas the red dots represent the 10^4 ^best parameter sets which were optimized. The resulting 2909 acceptable parameter sets that describe the experimental data are shown in green. Model predictions for the depositioning of hepatic triglycerides (C) and cholesterylesters (F) in cytoplasmic and endoplasmic reticulum fractions were obtained for all acceptable parameter sets. Note that only the total pools of triglyceride and cholesterylester were measured. The predictions for the triglyceride fractions are consistent, whereas the predictions for the cholesterylester fractions show a larger spread of possible outcomes.

A large-scale parameter estimation protocol was employed to capture multiple parameter sets that describe the experimental data of phenotype A (wild-type C57BL/6J mice). Mass isotopomer distribution analyses indicate that the metabolic fluxes are expected to be in the *μ*M/h range [38,39]. Therefore, parameter sets corresponding to unphysiologically high fluxes for any of the reactions (*>*100 mM/h) were removed from further analyses. Finally, a collection of 2909 acceptable parameter sets was obtained that describe the experimental data. With respect to the parameter values, it appeared that several are very constrained by the data and have a well defined value, whereas others show a larger spread of possible outcomes. Figure 2 shows an example of four parameter combinations, in which the black dots represent the initial sampled parameter sets, the red dots represent the ten thousand best parameter sets, and the green dots represent the optimized acceptable parameter sets that describe the experimental data. The observed variation in several parameters is reflected in specific model predictions. Figure 2 shows two examples of model predictions obtained for all acceptable parameter sets for the depositioning of hepatic triglycerides and cholesterylesters in cytoplasmic and endoplasmic reticulum fractions. Note that only the total pools of triglycerides and cholesterylesters were measured [29]. Nonetheless, the predictions for the triglyceride fractions are consistent, due to the data of triglyceride deposition and production rates in cytoplasmic and endoplasmic reticulum fractions [37]. However, the predictions for the cholesterylester fractions show a larger spread of possible outcomes. The latter case illustrates the importance of exploring differences between feasible parameter sets to assess the uncertainty associated with model predictions.

### Parameter adaptations from the wild-type to the LXR activated phenotype

**Figure 3:**
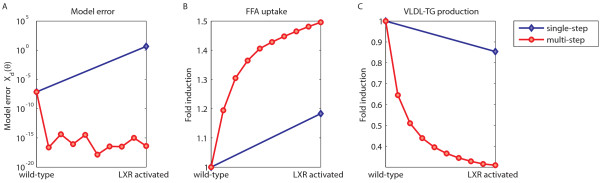
**Single-step versus multi-step optimization**. A) An example of an acceptable parameter set describing the wild-type phenotype, which was not successfully optimized by single-step optimization to describe the LXR activated phenotype. This problem was circumvented by multi-step optimization. In the latter case, the parameter estimation is carried out in a step-wise fashion. Hereto, the experimental data is interpolated from the wild-type phenotype to the LXR activated phenotype. At each interpolation step the parameters are reoptimized in order to describe the newly interpolated data. This procedure is repeated until the final state representing phenotype B is reached. B, C) Two examples of corresponding parameter trajectories from the wild-type phenotype to the LXR activated phenotype, normalized by the initial parameters of the wild-type phenotype.

Using the previously described techniques, an analysis was carried out to study the metabolic consequences of T0901317 induced LXR activation. It was assumed that metabolic adaptations upon LXR activation proceed linearly in time [40]. Therefore, a linear interpolation scheme was used for the step-wise optimization to describe the transition between phenotypes. A beneficial consequence of the approach is that the step-wise optimization guides the parameter estimation algorithm and hereby could overcome potential practical problems, such as convergence to local unacceptable minima. Figure 3 shows an example of an acceptable parameter set describing the wild-type phenotype, which was not successfully reoptimized by single-step optimization to describe the LXR activated phenotype, whereas this problem was circumvented by multi-step optimization.

**Figure 4:**
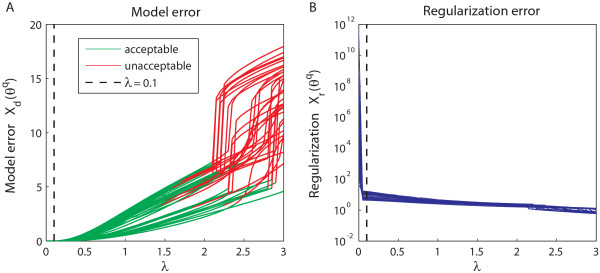
**Effect of regularization strength**. A) Model error *X_d _*for all acceptable parameter sets for increasing *λ*, where green indicates an acceptable data fit and red an unacceptable data fit. B) Regularization error *X_r _*for increasing *λ*. Note that a small *λ *is already sufficient to minimize parameter changes, while describing the experimental data still very well. A *λ *of 0.1 was selected (dashed line).

**Figure 5:**
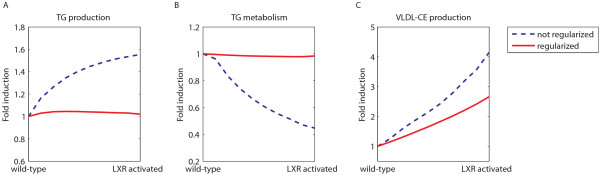
**Regularization of parameter trajectories**. Three examples of parameter trajectories from the wild-type phenotype to the LXR activated phenotype obtained without regularization (blue dashed) and with regularization using *λ *= 0.1 (red).

The parameter trajectories were regularized according to equation (6) to avoid needless change of parameters. A potential risk of regularization, as always with multi-objective optimization, is that for a low *λ *the regularization term has no effect, whereas for a large *λ *the parameter estimation algorithm might focus on minimizing the regularization term while describing the experimental data inaccurately. Therefore, the effect of *λ *on the sum of squared model errors *X_d _*and the sum of squared parameter differences *X_r _*was investigated for a collection of acceptable parameter sets. Figure 4a shows *X_d _*for increasing *λ*, where green indicates an acceptable data fit and red an unacceptable data fit. Figure 4b shows *X_r _*for increasing *λ*. Note that a small *λ *is already sufficient to minimize parameter changes, while the experimental data is still described very well. It is preferred to bias the data fitting as little as possible and therefore a *λ *of 0.1 was selected (both for the forward and backward trajectories). Figure 5 shows three examples of parameter trajectories from the wild-type phenotype to the LXR activated phenotype obtained without regularization (blue dashed) and with regularization (red). Both the regularized and unregularized parameter trajectories are acceptable in terms of model error *X_d_*. Note that the triglyceride production and metabolism parameters counteract each others effect and not necessarily have to change to describe the change in phenotype (Figure 5a,b). In some cases a less prominent change is sufficient to describe the change in phenotype (Figure 5c).

**Figure 6:**
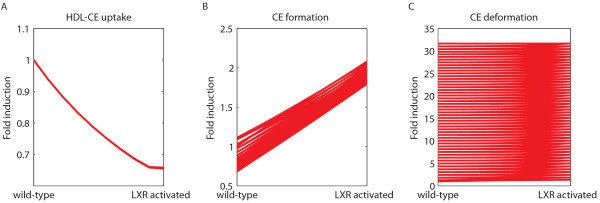
**(In)consistency of parameter adaptation trajectories**. Three examples of back and forward parameter trajectories from the wild-type phenotype to the LXR activated phenotype, using a hundred repetitions.

The uncertainty associated with parameter trajectories was investigated, among other things, by repeatedly calculating forward and backward trajectories. Figure 6 shows three examples of back and forward parameter trajectories from the wild-type phenotype to the LXR activated phenotype, using a hundred repetitions. Some parameters change consistently (Figure 6a,b), whereas others show a large spread in possible outcomes (Figure 6c).

**Figure 7:**
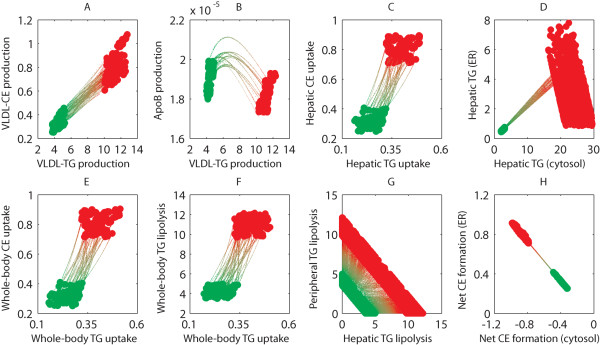
**Flux adaptations upon T0901317 induced LXR activation**. Flux trajectories from the wild-type phenotype (green) to the LXR activated phenotype (red). The data interpolation was carried out in a hundred steps and the back and forward flux trajectories were determined using a hundred repetitions. Molecular fluxes (A-C, E-H) are given in mM/h, whereas the triglyceride concentrations presented in (D) are given in mM.

The parameter adaptation trajectories were determined for all acceptable parameter sets and used to determine how the fluxes of triglycerides and cholesterols change in time from the wild-type phenotype to the LXR activated phenotype. The data interpolation was carried out in a hundred steps and the back and forward flux trajectories were determined using a hundred repetitions. Figure 7 shows pairs of flux trajectories of several metabolic processes included in the model, where the large green and red dots respectively represent the wild-type phenotype and the LXR activated phenotype. The small dots represent the artificial intermediate phenotypes. The majority of these flux trajectories are reproduced very consistently for the different parameter sets. The main findings are that both the VLDL-TG and VLDL-CE production are increased (Figure 7a), whereas the production of apolipoprotein B is slightly decreased (Figure 7b). The hepatic and whole-body uptake of triglycerides and cholesterols are increased (Figure 7c, e, and 7f). The increased hepatic triglyceride fluxes are especially stored in cytosolic fractions, rather than in endoplasmic reticulum fractions (Figure 7d). Furthermore, the net synthesis of cholesterylester from endogenous free cholesterol is decreased in the cytosol, yet increased in the endoplasmic reticulum (Figure 7h).

**Figure 8:**
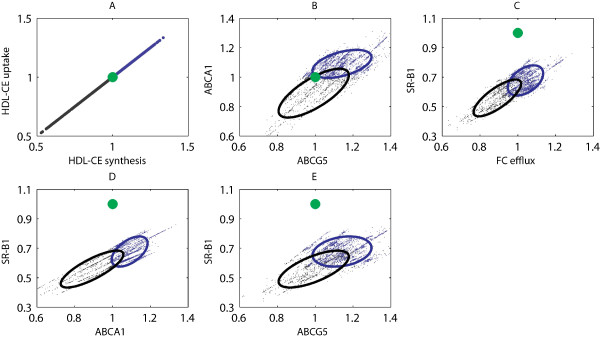
**Adaptations in cholesterol metabolism upon T0901317 induced LXR activation**. Adaptations in metabolic processes/components involved in hepatic cholesterol metabolism, normalized by the values of corresponding wild-type phenotype. The green dots represent the wild-type phenotype, whereas the blue and black dots represent the LXR activated phenotype. Solutions corresponding to an increased HDL-CE synthesis/uptake rate are colored blue, whereas solutions corresponding to a decreased HDL-CE synthesis/uptake rate are colored black. The ellipses were calculated by means of principal component analysis (PCA) and contain 95% of the corresponding solutions. ABCA1, ATP-binding cassette transporter 1; ABCG5, ATP-binding cassette transporter G5; SR-B1, scavenger receptor class B1.

As described in previous sections, several parameters are not constrained by the data and show a large spread of possible outcomes. This makes the calculation of consistent quantitative trajectories impossible. Nonetheless, relative changes with respect to the initial values of the wild-type phenotype could still provide useful information, e.g., to determine ranges of feasible fold inductions of molecular concentrations and fluxes, and to discriminate between different possible scenarios. The latter could be used to generate new hypotheses and to guide the design of new experiments. An example is depicted in Figure 8, showing adaptations in metabolic processes/components involved in hepatic cholesterol metabolism, normalized by the values of corresponding wild-type phenotype. The green dots represent the wild-type phenotype, whereas the blue and black dots represent the LXR activated phenotype. A positive correlation between HDL-CE synthesis and HDL-CE uptake by the liver was observed (Figure 8a). Both fluxes are either increased or decreased depending on the chosen parameter set. To investigate how these different scenarios are reflected in other related metabolic processes, solutions corresponding to an increased HDL-CE synthesis/uptake rate are colored blue, whereas solutions corresponding to a decreased HDL-CE synthesis/uptake rate are colored black. Different clusters of possible scenarios exist depending on how the HDL-CE synthesis/uptake rate adapts. The ellipses were calculated by means of principal component analysis (PCA) and contain 95% of the corresponding solutions.

## Discussion

To improve our understanding of progressive diseases such as diabetes mellitus and metabolic syndrome, the study of phenotype transitions is important. A challenging task is to explain the progression of phenotype transitions in terms of molecular adaptations in underlying biological systems. Here, we propose a novel computational approach to analyze biological systems evolving through various phenotypes and to predict which molecular processes are responsible for the transition. We presented a case involving the activation of the liver X receptor, a promising drug target for atherosclerotic therapies.

### Parameter adaptation trajectories during phenotype transitions: strengths and limitations

A large-scale parameter estimation protocol was employed to capture multiple parameter sets describing the biological system of phenotype A. A collection of 2909 acceptable parameter sets were obtained that describe the experimental data. A substantial fraction of the optimized parameter sets were not acceptable. These parameter sets did either not describe the experimental data or displayed unphysiologically high fluxes for any of the reactions. It appeared that the latter criterion contributed significantly to the rejection of parameter sets. The efficiency of sampling acceptable parameter sets could potentially be improved by including the rejection criteria in the optimization objective function. Note, that the computational approach is not restricted to the parameter estimation protocol presented here. Various parameter optimization methods exist that minimize the difference between experimental data and corresponding model simulations, e.g., trust-region optimization methods, simulated annealing, and genetic algorithms [31]. All these methods have their own merits and shortcomings, and therefore the preference for a certain protocol varies on a case-by-case basis.

Parameter trajectories describing the phenotype transition were determined by interpolating the data between phenotypes. The data interpolation was carried out in a hundred steps. We have performed several tests by using different numbers of steps. Performing more than a hundred steps did not change the results significantly. The computational approach allows free choice of interpolation schemes. Hence, when information is available about the progressive nature of certain biological processes, this information could be incorporated in the interpolation scheme. Furthermore, the computational approach could be used to explore different possible transition scenarios by employing a variety of different interpolation schemes. The latter could be useful when sufficient information about the transition characteristics is lacking, e.g., to test hypotheses about the feasibility of specific transitions with respect to the available experimental data. In this work we focused on diseases that arise progressively, e.g., hepatic steatosis, diabetes type 2, and metabolic syndrome. However, some diseases arise abruptly like in case of diabetes type 1. In latter cases it could be relevant to explore switch-like interpolation schemes and investigate whether the computational model can exhibit bistable behavior [41-45]. Here, it has been assumed that metabolic adaptations upon LXR activation proceed linearly in time. Although there is limited information about the dynamic response upon T0901317 induced LXR activation, this assumption is supported by experimental observations from Okazaki et al. showing a fairly linear response in plasma triglyceride and cholesterol levels in wild-type and Ldlr *^-/- ^*mice treated with T0901317 [40]. Although initial and final points of the trajectories were consistent with experimental data, the actual trajectories between phenotypes partly depended on the selected interpolation scheme. If more time-course data of LXR activated C57BL/6J mice would be available, a more realistic interpolation scheme could be defined. Although the dynamic behavior of parameter trajectories depends on the selected interpolation scheme, the relation between parameter trajectories (as visualized in Figure 7) does not necessarily have to change for different interpolation schemes. Namely, in the case all measured metabolite concentrations/fluxes adapt in a similar way, i.e. it can be assumed that the interpolation scheme is identical for each measurement, the relation between parameter trajectories remains identical. The results depicted in Figure 7 were reproduced using a quadratic-like and inverse-quadratic-like interpolation scheme for the measurements (Additional file 1). To identify minimal parameter adaptations that are necessary to describe a phenotype transition, the parameter estimation protocol was extended by including a regularization term given by the sum of squared parameter changes. This prevents unnecessarily changes of parameter values. The strength of the regularization term, determined by *λ*, was chosen carefully. It is preferred to bias the data fitting as little as possible and therefore a minimal value for *λ*, while still being effective, was selected. From a physical point of view, the regularization term could be interpreted as a measure for the energy cost, e.g., in terms of ATP production, to achieve a certain system adaptation. In future research, the approach could be refined by introducing multiple *λ *parameters to take account for different energy costs for the various processes included in a model.

### Metabolic adaptations upon T0901317 induced LXR activation

A computational model of hepatic lipid and plasma lipoprotein metabolism was developed to study the metabolic consequences of LXR activation. We were able to quantitatively integrate data of wild-type and LXR activated C57BL/6J mice into a consistent model and identified trajectories of parameter adaptations, describing the change in phenotype. The presented model predictions are in good agreement with experimental observations by other groups and contribute to the current understanding of LXR activation. The VLDL-TG production rate increases about 2.6 times upon LXR activation, as predicted by the model and experimentally measured [29]. A novel finding is that model predictions indicate that the VLDL-CE production rate increases as well (2.5 fold induction) and hereby contributes to the increase of plasma cholesterol levels. Model predictions indicate that the production of apolipoprotein B decreases slightly, which was also observed by [29,30,46]. This is reflected in an increase of VLDL particle diameter (94 ± 12 nm to 129 ± 9 nm). A novel model prediction is that the liver plays a major role in the re-uptake of lipoproteins (2.5 fold induction) and hereby prevents plasma hyperlipidemia. This flux prediction was not directly measured, but is in good agreement with gene expression data showing increased hepatic levels of the VLDL and LDL receptor [29]. Interestingly, model predictions indicate that the uptake of lipoproteins at peripheral tissues is negligible. Model analysis reveals that the uptake of triglycerides through lipolysis by lipases is increased as well (2.6 fold increase), which is in correspondence with gene expression data showing a significant induction of the lipoprotein lipase gene [29,30,47]. A significant increase in hepatic triglyceride level (6.92 ± 2.65 versus 57.74 ± 16.61 nmol/mg liver) was observed by [29], which is partly caused by an induction of lipogenic genes [29,30,46-50]. A novel model prediction is that the increased triglyceride fluxes are especially stored in cytosolic fractions, rather than in endoplasmic reticulum fractions which are predominantly used for incorporation into nascent produced VLDL particles. The increased level of ER triglycerides is partly caused by stimulation of the mobilization of triglycerides from the cytosol to the ER. This is confirmed by several studies indicating that a large part of secreted VLDL triglycerides are derived via lipolysis of cytosolic triglyceride storage pools [51-55]. A relevant follow-up study would be to determine whether these differences are associated with alterations in diglyceride acyltransferase activities (DGAT1 and DGAT2), which play a crucial role in the biosynthesis and deposition of triglycerides [56-59]. Another interesting example which could guide the design of new experiments is depicted in Figure 8, showing adaptations in metabolic processes/components involved in hepatic cholesterol metabolism. Different clusters of possible scenarios exist depending on how the HDL-CE synthesis/uptake rate adapts. Hence, many solutions could potentially be excluded by measuring one of these fluxes/components. With respect to this, the 'blue' scenario is probably more plausible for several reasons. First, these solutions are associated with an increased level of the ATP-binding cassette transporter G5 (ABCG5), resulting in an increased biliary excretion of free cholesterol. Secondly, these solutions correspond to an increased level of the ABCA1 transporter, which is responsible for the efflux of cholesterol from peripheral tissues to HDL [30,47,49,50].

### Mathematical modeling of progressively adapting biological systems

Mathematical modeling is well suited for integrating different sources of experimental data for a certain phenotype and allows investigating of the complex interactions of underlying biological systems. A mathematical model can be used to obtain thorough understanding of a biological system, e.g. by investigating its complex behavior in response to various stimuli. However, simulating and predicting long-term progressive adaptations is challenging. The multiscale nature of progressively adapting biological systems complicates the development of predictive computational models. As such, one of the central and formidable challenges in systems biology is to develop multiscale computational models and methods that can be used to study molecular mechanisms underlying progressive diseases [60-65]. Furthermore, model parameters that determine the kinetics of molecular processes are often assumed to be constant in time and between phenotypes. This is most probably a valid assumption to study short-term processes, e.g., initial response kinetics to perturbations of a biological system. In case of progressively adapting biological processes, it is questionable whether this assumption still holds. For instance, effects of aging, changes in body composition, or other developmental changes, influence the phenotypical characteristics and the transition between phenotypes.

The computational approach presented here was employed to study the metabolic consequences of LXR activation, which displays several of the aforementioned issues. For example, the underlying biological system contains processes at timescales ranging from seconds to hours, whereas the phenotypical characteristics develop at a timescale ranging from days to weeks in mice. Our approach has as advantage that it can readily deal with large differences in timescales. For instance, long-term changes in short-term processes could be studied by explicitly modeling the short-term processes, whereas the long-term modulation could be captured by identifying necessary parameter changes. This implies that molecular adaptations could be described without the necessity to develop detailed kinetic models of the modulating mechanisms. This is a major advantage, e.g., for the LXR case, as LXRs modulate a wide range of heavily interlinked complex metabolic processes and signal transduction pathways of which the kinetics and molecular mechanisms are not well understood.

## Conclusions

The study of phenotype transitions is important to understand disease progression. We developed a novel computational approach to analyze molecular adaptations in a biological system evolving through various phenotypes, which is generically applicable to different biological systems. For the case of liver X receptor induced hepatic steatosis the novel approach yields information about the redistribution of fluxes and pools of triglycerides and cholesterols that was not directly apparent from the experimental data. The collection of parameter and corresponding flux trajectories give a broad overview of key-processes that are involved in the phenotype transition and how they potentially change in time. Model analysis provides guidance which specific molecular processes to study in more detail to obtain further understanding of the underlying biological system. The main findings are that both the VLDL-TG and VLDL-CE production rates are increased, as well as the uptake of triglycerides through lipolysis. The liver plays a major role in the re-uptake of lipoproteins and hereby prevents plasma hyperlipidemia. The increased triglyceride fluxes are especially stored in cytosolic fractions, rather than in endoplasmic reticulum fractions.

## Authors' contributions

CT developed the mathematical model, performed the computational analysis, and wrote the paper. JV contributed to the computational analysis, improved the computational efficiency of the software, and revised the paper. PH supervised the study and revised the paper. NR supervised the study, contributed to the computational analysis, and revised the paper. All authors read and approved the final manuscript.

## Supplementary Material

Additional file 1 **Supplementary material**. Description of model equations, additional analyses, implementation details, and experimental data. Click here for file

Additional file 2 **SBML file**. Implementation of the mathematical model in SBML format. Click here for file
