# Supplementary material for: Parameter adaptations during phenotype transitions in progressive diseases
Source: BMC Syst Biol. 2011 Oct 26;5:174. doi: 10.1186/1752-0509-5-174 (PMC3354367; doi:10.1186/1752-0509-5-174)
Supplement: Additional file 1 — Supplementary material. Description of model equations, additional analyses, implementation details, and experimental data. [file 1752-0509-5-174-S1.PDF]

Supplementary material

Experimental data

Several data sets of wild-type and T0901317 LXR activated C57BL/6J mice were used for model parameterization (Table 1). The unit mmol/L was used to describe molecular concentrations in the mathematical model. The experimental data of hepatic species from [1], which were given in nmol/mg liver, were converted, assuming a hepatic volume of 2.5 mL [2]. Furthermore, the experimental data of cholesterol fluxes from [3], which were given in mg/day/kg, were converted, assuming a molecular weight of 647.9 g/mol [4].

Calculation of VLDL particle diameter

The diameter of nascent produced VLDL particles was calculated as follows. As each VLDL particle contains one apolipoprotein B particle, the number of triglyceride and cholesterylester molecules per VLDL particle can be determined by dividing corresponding fluxes by the total number of apolipoprotein B proteins. Subsequently, the core volume of a VLDL particle was determined assuming a molecular volume of 946.84 mL/mol for triglyceride and a molecular volume of 685.48 ml/mol for cholesterylester [4]. A core radius was calculated from the core volume assuming a spherical shape of the VLDL particles. Furthermore, the particle membrane accounts for an additional two nanometers [5].

Model equations

The mathematical model contains eight molecular species x (Table 2) interlinked by twenty-four flux interactions (Table 3). The ordinary differential equations are given by:

$$
\begin{aligned}
d\dot{x}_{FC} &= F_{FC_{prod}} - F_{FC_{met}} - F_{CEfor_{cyt}} + F_{CEdef_{cyt}} - F_{CEfor_{ER}} + F_{CEdef_{ER}} \\
d\dot{x}_{CE_{cyt}} &= F_{CEfor_{cyt}} - F_{CEdef_{cyt}} + \frac{V_{plasma}}{V_{hep}} \left(F_{CEupt_{hep}} + F_{CEupt_{HDL}} \right) \\
d\dot{x}_{CE_{ER}} &= F_{CEfor_{ER}} - F_{CEdef_{ER}} - F_{VLDL-CE} \\
d\dot{x}_{TG_{cyt}} &= F_{TGprod_{cyt}} - F_{TGmet_{cyt}} + F_{TGfor_{cyt}} - F_{TGfor_{ER}} + \frac{V_{plasma}}{V_{hep}} \left(F_{FFA} + F_{TGupt_{hep}} + F_{TGhyd_{hep}} \right) \\
d\dot{x}_{TG_{ER}} &= F_{TGprod_{ER}} - F_{TGmet_{ER}} + F_{TGfor_{ER}} - F_{TGfor_{cyt}} - F_{VLDL-TG} \\
d\dot{x}_{TG_{plasma}} &= \frac{V_{hep}}{V_{plasma}} F_{VLDL-TG} - F_{TGupt_{hep}} - F_{TGupt_{per}} - F_{TGhyd_{hep}} - F_{TGhyd_{per}} \\
d\dot{x}_{CE_{plasma}} &= \frac{V_{hep}}{V_{plasma}} F_{VLDL-CE} - F_{CEupt_{hep}} - F_{CEupt_{per}} \\
d\dot{x}_{CE_{HDL}} &= F_{CEfor_{HDL}} - F_{CEupt_{HDL}}
\end{aligned}
$$

Where V_{hep} and V_{plasma} respectively represent the compartment volumes of the liver and blood plasma.

Implementation

The mathematical model and parameter estimation routines were implemented in Matlab (2007b, The MathWorks, Natick, Massachusetts). The ordinary differential equations were solved using the variable-order multistep solver CVODES from Sundials (2.6.0, Lawrence Livermore National Laboratory, Livermore, California) [6]. A relative error tolerance of 10^{-8} and an absolute error tolerance of 10^{-10} were used. The Matlab nonlinear least-squares solver LSQNONLIN, which uses an interior reflective Newton

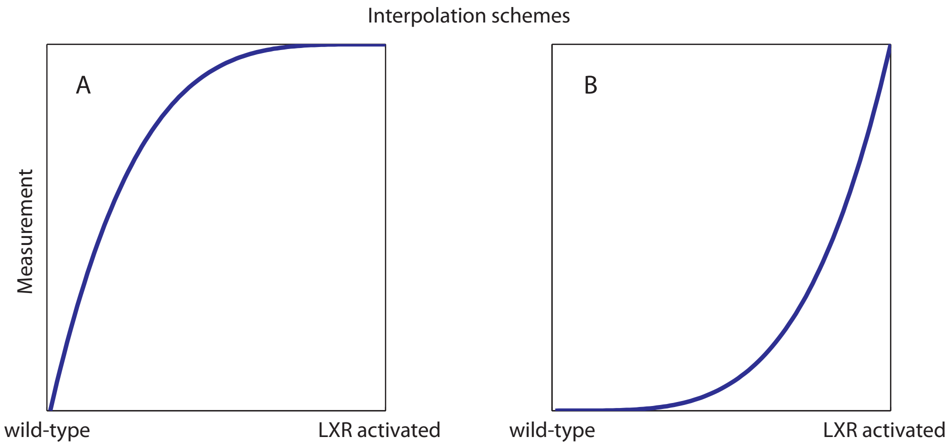

Figure 1. Two different interpolation schemes that were used to model the transition between phenotypes. The results obtained with interpolation scheme A are depicted in Figure 2, whereas the results obtained with interpolation scheme B are depicted in Figure 3.

method, was used to estimate model parameters [7]. Both the termination tolerances for the objective function and the parameter estimates were set to 10^{-8}.

Parameter trajectories with different interpolation schemes

In the main text, parameter adaptation trajectories were calculated using a linear interpolation scheme. Although the dynamic behavior of parameter trajectories depends on the selected interpolation scheme, the relation between parameter trajectories (as visualized in Figure 7 in the main text) does not necessarily have to change for different interpolation schemes. Namely, in the case all measured metabolite concentrations/fluxes adapt in a similar way, i.e. it can be assumed that the interpolation scheme is identical for each measurement, the relation between parameter trajectories remains identical. The results depicted in Figure 7 were reproduced using a quadratic-like and inverse-quadratic-like interpolation scheme (Figure 1) for the measurements. The result are respectively depicted in Figure 2 and 3.

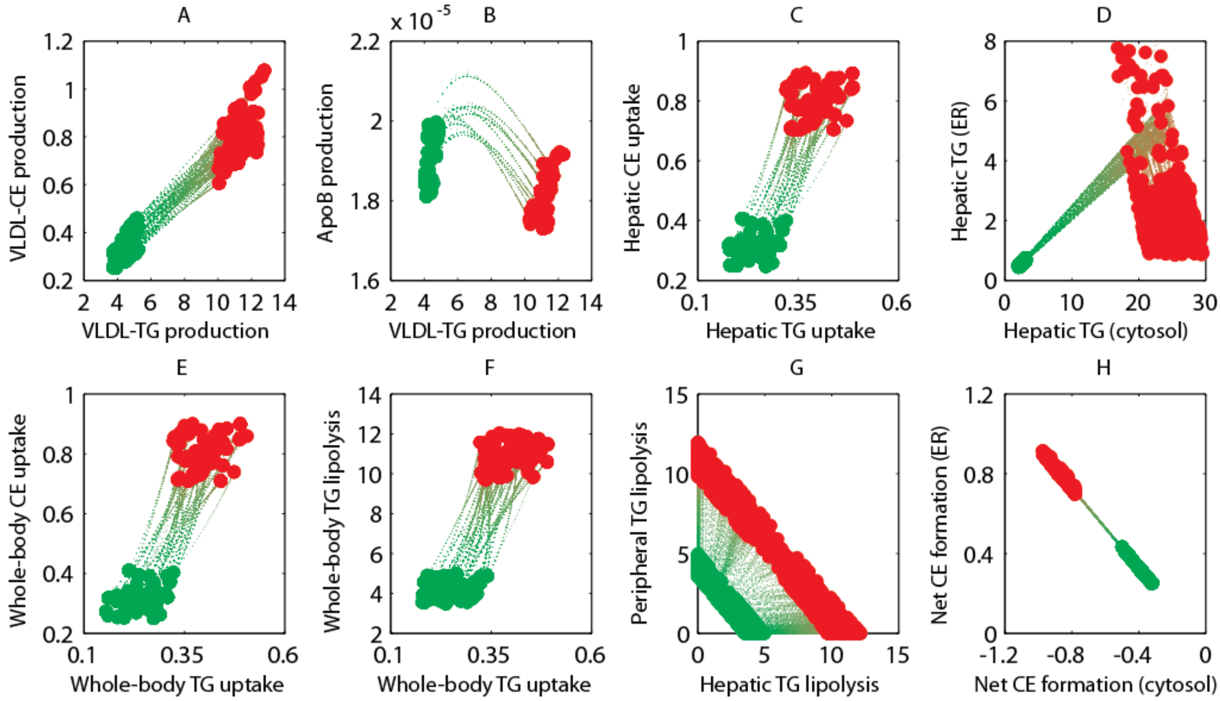

Figure 2. Flux trajectories from the wild-type phenotype (green) to the LXR activated phenotype (red). Molecular fluxes (A-C,E-H) are given in mM/h, whereas the triglyceride concentrations presented in (D) are given in mM. The interpolation scheme depicted in Figure 1 A was used to calculate the trajectories.

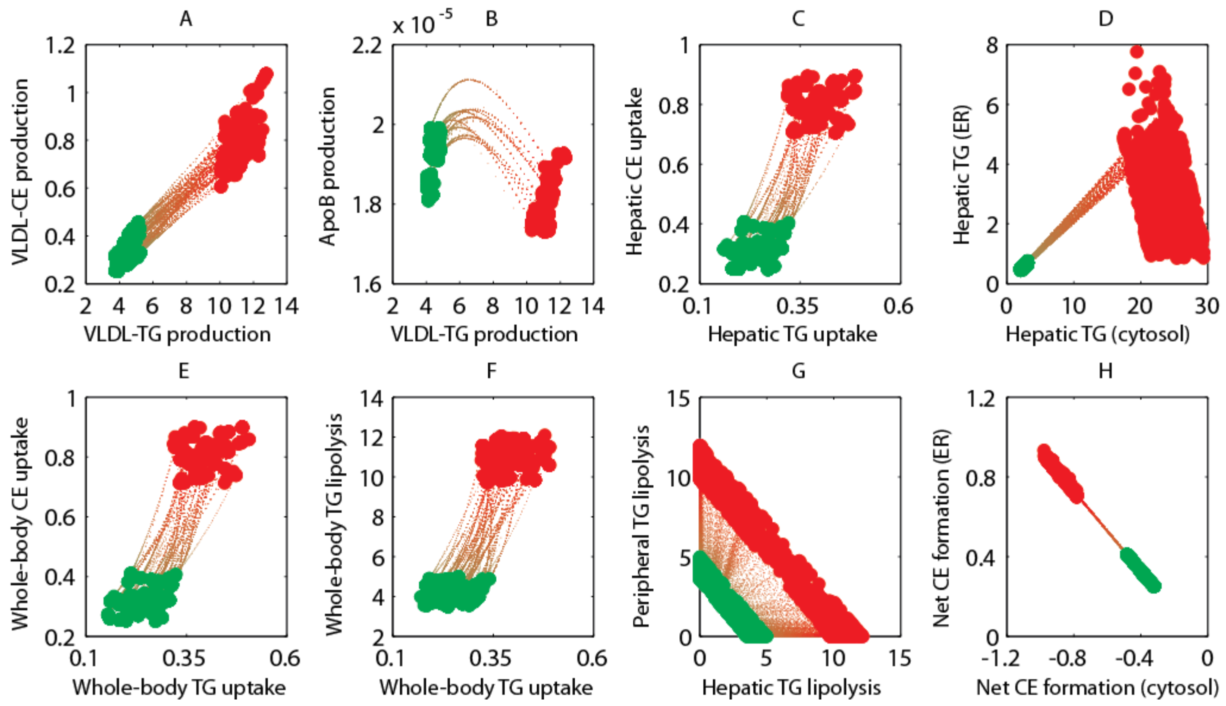

Figure 3. Flux trajectories from the wild-type phenotype (green) to the LXR activated phenotype (red). Molecular fluxes (A-C,E-H) are given in mM/h, whereas the triglyceride concentrations presented in (D) are given in mM. The interpolation scheme depicted in Figure 1 B was used to calculate the trajectories.

Data	Value	Unity	Reference
wild-type			
hepatic triglyceride	6.92 ± 2.65	nmol/mg liver	[1]
hepatic free cholesterol	4.21 ± 0.50	nmol/mg liver	[1]
hepatic cholesterylester	1.44 ± 0.52	nmol/mg liver	[1]
plasma free cholesterol	0.60 ± 0.07	mM	[1]
plasma cholesterylester	1.36 ± 0.31	mM	[1]
HDL cholesterol	1.35 ± 0.27	mM	[1]
plasma triglyceride	0.46 ± 0.16	mM	[1]
free fatty acids	0.34 ± 0.07	mM	[1]
VLDL-TG production	78.8 ± 18.7	μmol/kg/h	[1]
VLDL diameter	94 ± 12	nm	[1]
VLDL free cholesterol	1.50 ± 0.31	mM	[1]
VLDL cholesterylester	0.63 ± 0.10	mM	[1]
VLDL triglycerides	28.53 ± 5.86	mM	[1]
hepatic free cholesterol production	34	mg/day/kg	[3]
hepatic HDL cholesterol uptake	34	mg/day/kg	[3]
peripheral cholesterol uptake	106	mg/day/kg	[3]
ratio TG (cytosol vs. ER)	4.48 ± 0.46	-	[8]
ratio TG production (cytosol vs. ER)	1.38 ± 0.27	-	[8]
T0901317 induced LXR activation			
hepatic triglyceride	57.74 ± 16.61	nmol/mg liver	[1]
hepatic free cholesterol	3.83 ± 0.71	nmol/mg liver	[1]
hepatic cholesterylester	0.98 ± 0.40	nmol/mg liver	[1]
plasma free cholesterol	0.87 ± 0.05	mM	[1]
plasma cholesterylester	2.27 ± 0.21	mM	[1]
HDL cholesterol	2.15 ± 0.14	mM	[1]
plasma triglyceride	0.49 ± 0.21	mM	[1]
free fatty acids	0.47 ± 0.08	mM	[1]
VLDL-TG production	201.9 ± 36.4	μmol/kg/h	[1]
VLDL diameter	129 ± 9	nm	[1]
VLDL free cholesterol	2.01 ± 0.31	mM	[1]
VLDL cholesterylester	1.34 ± 0.19	mM	[1]
VLDL triglycerides	47.10 ± 8.66	mM	[1]

Table 1. Overview of experimental data of wild-type and T0901317 LXR activated C57BL/6J mice used for model parameterization.

State / input	Description
x_{FC}	hepatic free cholesterol
$x_{CE_{cyt}}$	hepatic cholesterylester (cytosol)
$x_{CE_{ER}}$	hepatic cholesterylester (endoplasmic reticulum)
$x_{TG_{cyt}}$	hepatic triglyceride (cytosol)
$x_{TG_{ER}}$	hepatic triglyceride (endoplasmic reticulum)
$x_{TG_{plasma}}$	plasma triglyceride
$x_{CE_{plasma}}$	plasma cholesterylester
$x_{CE_{HDL}}$	high-density-lipoprotein (HDL) cholesterylester
u	free fatty acid (model input)

Table 2. Overview and description of state variables and inputs.

Flux	Equation	Description
F_{FCprod}	p_{FCprod}	hepatic free cholesterol production
F_{FCmet}	$p_{FCmet} \times x_{FC}$	hepatic free cholesterol metabolism
$F_{CEfor_{cyt}}$	$p_{CEfor_{cyt}} \times x_{FC}$	hepatic cholesterylester synthesis (cytosol)
$F_{CEdef_{cyt}}$	$p_{CEdef_{cyt}} \times x_{CE_{cyt}}$	hepatic cholesterylester degradation (cytosol)
$F_{CEfor_{ER}}$	$p_{CEfor_{ER}} \times x_{FC}$	hepatic cholesterylester synthesis (endoplasmic reticulum)
$F_{CEdef_{ER}}$	$p_{CEdef_{ER}} \times x_{CE_{ER}}$	hepatic cholesterylester degradation (endoplasmic reticulum)
$F_{TGprod_{cyt}}$	$p_{TGprod_{cyt}}$	hepatic triglyceride production (cytosol)
$F_{TGmet_{cyt}}$	$p_{TGmet_{cyt}} \times x_{TG_{cyt}}$	hepatic triglyceride metabolism (cytosol)
$F_{TGfor_{cyt}}$	$p_{TGfor_{cyt}} \times x_{TG_{ER}}$	hepatic triglyceride transport to cytosol
$F_{TGprod_{ER}}$	$p_{TGprod_{ER}}$	hepatic triglyceride production (endoplasmic reticulum)
$F_{TGmet_{ER}}$	$p_{TGmet_{ER}} \times x_{TG_{ER}}$	hepatic triglyceride metabolism (endoplasmic reticulum)
$F_{TGfor_{ER}}$	$p_{TGfor_{ER}} \times x_{TG_{cyt}}$	hepatic triglyceride transport to endoplasmic reticulum
F_{FFA}	$p_{FFA} \times u$	hepatic uptake of free fatty acids
$F_{VLDL-TG}$	$p_{VLDL-TG} \times x_{TG_{ER}}$	very-low-density-lipoprotein (VLDL) triglyceride production
$F_{VLDL-CE}$	$p_{VLDL-CE} \times x_{CE_{ER}}$	very-low-density-lipoprotein (VLDL) cholesterylester production
$F_{TGupt_{hep}}$	$p_{upt_{hep}} \times x_{TG_{plasma}}$	hepatic uptake of triglycerides
$F_{CEupt_{hep}}$	$p_{upt_{hep}} \times x_{CE_{plasma}}$	hepatic uptake of cholesterylesters
$F_{TGupt_{per}}$	$p_{upt_{per}} \times x_{TG_{plasma}}$	peripheral uptake of triglycerides
$F_{CEupt_{per}}$	$p_{upt_{per}} \times x_{CE_{plasma}}$	peripheral uptake of cholesterylesters
$F_{CEfor_{HDL}}$	$p_{CEfor_{HDL}}$	high-density-lipoprotein (HDL) cholesterylester synthesis
$F_{CEupt_{HDL}}$	$p_{CEupt_{HDL}} \times x_{CE_{HDL}}$	hepatic uptake of high-density-lipoprotein (HDL) cholesterylesters
$F_{TGhyd_{hep}}$	$p_{TGhyd_{hep}} \times x_{TG_{plasma}}$	hepatic uptake of triglycerides via lipase hydrolysis
$F_{TGhyd_{per}}$	$p_{TGhyd_{per}} \times x_{TG_{plasma}}$	peripheral uptake of triglycerides via lipase hydrolysis

Table 3. Overview and description of model fluxes.

References

[1] Grefhorst A, Elzinga B, Voshol P, Plösch T, Kok T, Bloks V, van der Sluijs F, Havekes L, Romijn J, Verkade H, et al.: **Stimulation of lipogenesis by pharmacological activation of the liver X receptor leads to production of large, triglyceride-rich very low density lipoprotein particles**. *J Biol Chem* 2002, **277**(37):34182–34190.

[2] Scheller L, Wirtz R, Azad A: **Susceptibility of different strains of mice to hepatic infection with Plasmodium berghei.** *Infect Immun* 1994, **62**(11):4844–4847.

[3] Osono Y, Woollett L, Herz J, Dietschy J: **Role of the low density lipoprotein receptor in the flux of cholesterol through the plasma and across the tissues of the mouse.** *J Clin Invest* 1995, **95**(3):1124–1132.

[4] Teerlink T, Scheffer P, Bakker S, Heine R: **Combined data from LDL composition and size measurement are compatible with a discoid particle shape**. *J Lipid Res* 2004, **45**(5):954–966.

[5] Miller A, Smith L: **Activation of lipoprotein lipase by apolipoprotein glutamic acid**. *J Biol Chem* 1973, **248**(9):3359–3362.

[6] Hindmarsh A, Brown P, Grant K, Lee S, Serban R, Shumaker D, Woodward C: **SUNDIALS: Suite of nonlinear and differential/algebraic equation solvers**. *ACM T Math Software* 2005, **31**(3):363–396.

[7] Coleman T, Li Y: **An Interior Trust Region Approach for Nonlinear Minimization Subject to Bounds**. *SIAM J Optimiz* 1996, **6**:418–445.

[8] Millar J, Stone S, Tietge U, Tow B, Billheimer J, Wong J, Hamilton R, Farese Jr R, Rader D: **Short-term overexpression of DGAT1 or DGAT2 increases hepatic triglyceride but not VLDL triglyceride or apoB production**. *J Lipid Res* 2006, **47**(10):2297–2305.
